# Effect of Sleep Deprivation on the Working Memory-Related N2-P3 Components of the Event-Related Potential Waveform

**DOI:** 10.3389/fnins.2020.00469

**Published:** 2020-05-19

**Authors:** Ziyi Peng, Cimin Dai, Yi Ba, Liwei Zhang, Yongcong Shao, Jianquan Tian

**Affiliations:** ^1^School of Psychology, Beijing Sport University, Beijing, China; ^2^Institute of Psychology, Chinese Academy of Sciences, Beijing, China; ^3^Naval Special Forces Recuperation Center, Qingdao, China

**Keywords:** sleep deprivation, working memory, event related potentials, electroencephalography, n-back

## Abstract

Working memory is very sensitive to acute sleep deprivation, and many studies focus on the brain areas or network activities of working memory after sleep deprivation. However, little is known about event-related potential (ERP)-related changes in working memory after sleep loss. The purpose of this research was to explore the effects of 36 h of total sleep deprivation (TSD) on working memory through ERPs. Sixteen healthy college students performed working memory tasks while rested and after 36 h of TSD, and electroencephalography (EEG) data were simultaneously recorded while the subjects completed working memory tasks that included different types of stimulus materials. ERP data were statistically analyzed using repeated measurements analysis of variance to observe the changes in the working memory-related N2-P3 components. Compared with baseline before TSD, the amplitude of N2-P3 components related to working memory decreased, and the latency was prolonged after TSD. However, the increased amplitude of the P2 wave and the prolonged latency were found after 36 h of TSD. Thus, TSD can impair working memory capacity, which is characterized by lower amplitude and prolonged latency.

## Introduction

With the progress of society and changes in work rhythm, an increasing number of people are suffering from sleep deprivation. Sleep deprivation not only damages the physical and mental health of the individual but also seriously affects work performance, causing work errors and even accidents. Therefore, understanding the mechanism of sleep deprivation that affects cognitive function is of great significance for effectively preventing the effects of sleep deprivation.

Previous studies have revealed that sleep deprivation can cause a series of changes in an individual’s mood, cognitive ability, work performance, and immune function (Choo et al., 2005). The lack of sleep disrupts body circulation and affects the cognitive and emotional abilities of individuals (Raymond, 1988). Several studies have revealed that sleep deprivation impairs response inhibition (Harrison and Horne, 1998; Muzur et al., 2002; Jennings et al., 2003). For example, after 36 h of sleep deprivation, the individual’s ability to suppress negative stimuli decreased (Chuah et al., 2006). Neuroimaging studies have suggested that sleep deprivation reduces an individual’s low-level of visual processing ability (Anderson and Platten, 2011; Ning et al., 2014). In addition, sleep deprivation impairs the hippocampus and could affect memory by destroying synaptic plasticity (Cote et al., 2014). Thomas (2003) has indicated that lack of sleep reduced cerebral blood flow and metabolic rate in the thalamus, prefrontal cortex, and parietal cortex (Géraldine et al., 2005). Jarraya and colleagues found that partial sleep deprivation significantly affected neuropsychological functions such as verbal instant memory, attention, and alertness (Thomas, 2003). Furthermore, some studies have revealed that the cumulative effects of partial sleep deprivation could severely impair cognitive function and behavior (Van Dongen, 2004; Scott et al., 2006; Jarraya et al., 2013).

Working memory is a system that used to store and process information and which is a cognitive function with limited capacity (Bartel et al., 2004). Moreover, the information stored in the working memory system can be changed from short-term memory to long-term memory through retelling and other memory methods. Working memory is the transition between short-term and long-term memory systems, which is very pivotal in human message processing (Miyake and Shah, 1999). It provides a temporary storage space and the resources needed to process information, such as voice understanding, reasoning, and learning. Sleep deprivation has been shown to affect working memory first.

Previous studies have used the n-back working memory paradigm in participants who underwent sleep deprivation and found that lack of sleep induces a decrease in metabolic activity in the brain’s regional network, which is mainly effected information processing and reaction inhibition (Baddeley, 2000; Zhang et al., 2019). Impaired working memory after sleep deprivation is related to the activation of the default network in tasks (Chee and Chuah, 2008), which may be related to the important role of the thalamus in cortical alertness. For instance, sleep deprivation increased the connection between the hippocampus, thalamus, and default network, which was often accompanied by higher subjective drowsiness and worse performance of working memory (Lei et al., 2015; Li et al., 2016). Studies on sleep deprivation identi?ed that increased latency and reduced amplitude of the P3 component were associated with prolonged sobriety (Morris et al., 1992; Jones and Harrison, 2001; Panjwani et al., 2010). The decrease in the P3 wave might reflect a decrease in participants’ attention and a reduction in the discernment of target stimuli (Koslowsky and Babkoff, 1992).

However, few studies have provided electrophysiological evidence for impaired working memory after sleep deprivation. The n-back task is considered a common method to assess working memory (Owen et al., 2005; Jaeggi et al., 2010). Zhang et al. designed a two-back pronunciation working memory task to explore the decreased message alternate of working memory during sleep deprivation, but few studies have used different types of working memory tasks in a single experiment. In the present study, we designed different types of working memory tasks (pronunciation working memory, spatial working memory, and object working memory) to explore the impairment of cognitive function by TSD and recorded participant EEG data at 2 time points (baseline and 36 h-TSD). All of the tasks adopted a 2-back paradigm. This study evaluated the changes in the N2-P3 wave related to working memory during TSD and analyzed the temporal characteristics of the effects of sleep deprivation on working memory. Our findings provide experimental evidence for the effects of sleep deprivation on cognitive function.

## Materials and Methods

### Participants

Sixteen young, healthy, right-handed male students participated in this study. We recruited participants by advertising on the campus. The participants all had good sleep habits (PSQI<5). All participants were aged between 21 and 28 years with an average age of 23 years, and none of the participants had any mental or physical illness. All participants had normal vision or corrected vision above 1.0 and intelligence scores >110 on the Raven Test. Before the experiment, the experimenter explained the procedure and points for attention to the participants to make sure they were familiar with the method and procedure. In the 2 weeks before the experiment, the participants slept regularly for 7–9 h per day, without smoking, drinking coffee, drinking alcohol, or consuming any medication for 2 days before the experiment. Before the experiment, all the participants provided written informed consent. The experimental scheme was approved by the Ethics Committee of the Fourth Military Medical University and Beihang University.

### Experimental Design

**FIGURE 1:**
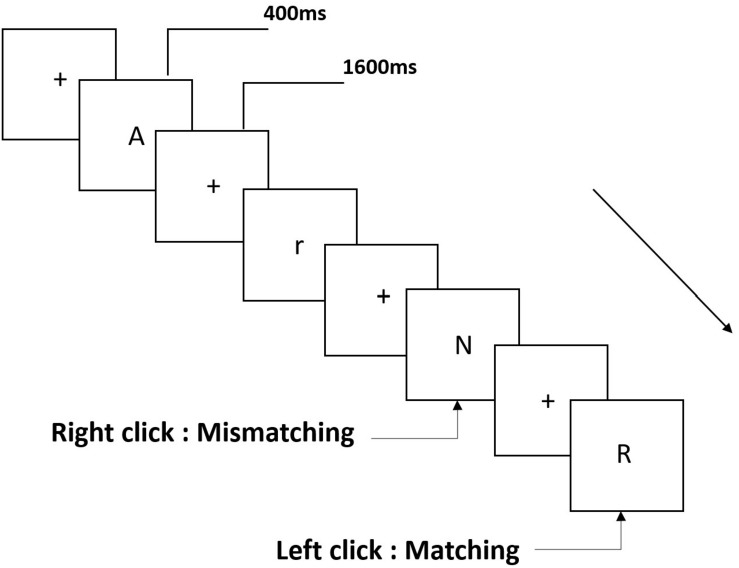
Schematic diagram of the pronunciation working memory task.

**FIGURE 2:**
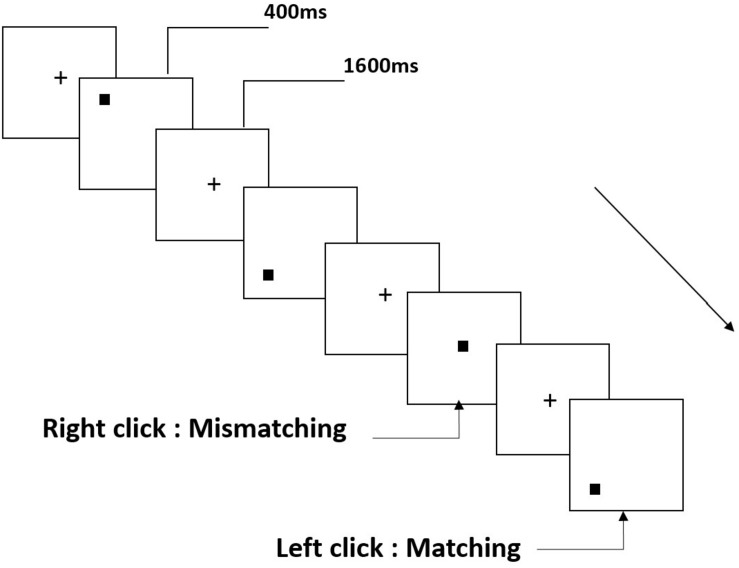
Schematic diagram of the spatial working memory task.

**FIGURE 3:**
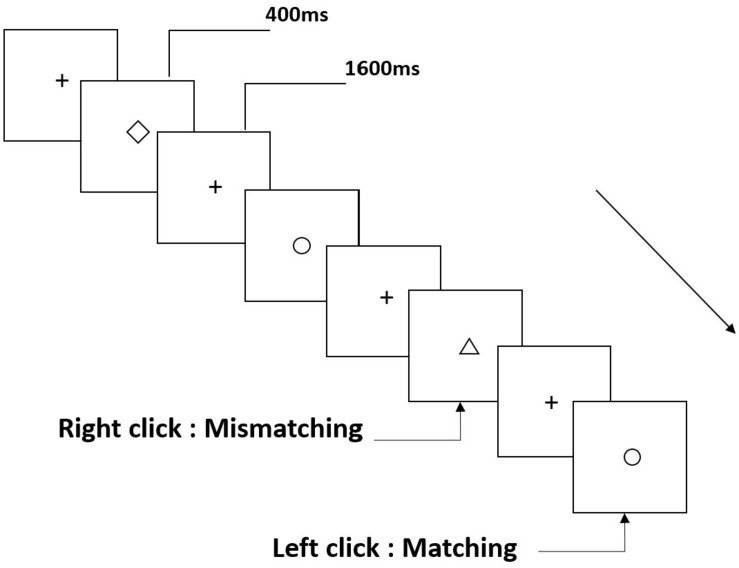
Schematic diagram of the object working memory task.

Three types of working memory tasks were presented to all participants. They were two-back pronunciation working memory task (see Figure 1), two-back spatial working memory task (see Figure 2), and two-back object working memory task (see Figure 3). The stimulate materials of the tasks were 15 case-insensitive English letters that excluding the ones with similar letters, such as L/l, M/m; small black squares; and 12 geometric figures, respectively. All of the materials were shown in black color on a white background, with an approximate visual angle of 1.5° × 1.5° (width: 2.0 cm, height: 2.0 cm) subtending. 122 trails were comprised in each task and, in each trail, the target stimulus was presented for 400 ms two trails after the presentation of objective stimulus, with the 1,600 ms stimulus onset asynchrony time (SOA) that was marked by a white “+.”The participants were asked to click the left mouse button when the target and objective stimulus were the same (“matching”), while click the right mouse button when they were not (“mismatching”). The matching or not condition were presented in a pseudorandom order with a 1:1 ratio.

### Experimental Procedures

Before the experiment, the participants were instructed of the experimental task. They were informed to practice the three types of working memory tasks until an accuracy rate of 90% was achieved. Participants visited the laboratory the day before the experiment and slept in the laboratory that night. The two partner participants performed the experiments at the same time. Three types of working memory tasks were performed at 7:30 am to 8:30 am the next morning with simultaneous electroencephalogram (EEG) recording (baseline). The second EEG recording (36 h-TSD) was conducted after a 36-h period during which the participants were not allowed to sleep. During the entire experiment time, central inhibition and stimulant drugs were forbidden. The participants were accompanied, observed and reminded by nursing staff in order to keep them awake throughout the TSD session.

### EEG Recordings

A continuous scalp EEG was recorded using electrode caps placed in 64 locations using the 10–20 system with a SynAmps2 amplifier. The bilateral mastoids (A1 and A2) were used for reference, and the forehead was used as a ground. EEGs were recorded at 1,000 Hz, and the impedance of all channels was maintained below 5 kΩ. Four additional electrodes were placed above and below the right and left eyes to record a bipolar vertical and horizontal electrooculogram.

### Data Analysis of Behavioral Experiments

Due to technical errors, two cases were deleted while other 14 cases were included in the following statistical analysis. Behavioral data included the mean reaction time, correct rate and the correct number per unit time. Behavioral data in baseline and 36 h-TSD states were recorded for analyzing. The analyses were run by IBM SPSS (V22.2), where the repeated measures analysis of variance (ANOVA) method with Greenhouse-Geisser was Bonferroni *post-hoc* analysis were launched. The statistical results were presented as the mean and standard deviation (SD).

### EEG Data Analysis

Scan 4.3 program was used to analyze the EEG data, where the EEG artifacts of the eye movement were corrected by ocular artifact reduction method. Epochs ranging from -100 to 800 ms of the continuous EEG data were extracted and filtered by a bandpass filter from 0.5 to 30 Hz with the frequency slope of 24 dB/oct. The trials in which the voltage exceeded ± 100 μV were rejected and the baseline was corrected to a mean amplitude of 100 ms. The EEG components were averaged and calculated with only the corrected responses. The ERP components P2 (100–250 ms), N2 (150–350 ms), and P3 (250–450 ms) of the stimulus trials were identified and quantified. The grand-average peak amplitudes and latencies of the N2 and P3 components were calculated separately at F3, Fz, F4, C3, Cz, C4, P3, Pz, and P4, and the P2 component was calculated at F3, Fz, F4, C3, Cz, and C4 (Casement et al., 2006; Verweij et al., 2014).

Repeated measures ANOVA was employed for all ERP results. The main effects and the interactions between sleep states (baseline and 36 h-TSD), tasks (pronunciation working memory, spatial working memory, and object working memory), regions (frontal, central, and parietal; the P2 component was analyzed only on the frontal and central regions), and sites (left, middle, and right) were statistically analyzed employing repeated measures ANOVA, which included Greenhouse-Geisser corrections for non-sphericity and Bonferroni *post-hoc* tests.

## Results

### Behavioral Performance

The results of the behavioral experiments are shown in Table 1. The mean reaction time was longer in the 36 h-TSD state than at baseline with a trend to increase [*F*_(1, 13)_ = 2.563, *P* = 0.133] but without significant differences. ANOVA revealed that the correct rate of the task was significantly different between the baseline and 36 h-TSD [*F*_(1, 13)_ = 10.153, *P* = 0.007]. The correct number per unit time showed a significant main effect of time during 36 h-TSD [*F*_(1, 13)_ = 7.010, *P* = 0.020].

**TABLE 1:** Performance data (mean ± SD) on the 2-back task at baseline and after 36 h-TSD.

	Baseline	36 h-TSD
Mean Reaction time (ms)	507.26 (82.04)	542.77 (103.73)
Correct rate (%)	0.94 (0.04)	0.85 (0.12)*
Correct number/sec	1.91 (0.35)	1.64 (0.42)*

*P < 0.05, vs. baseline.

### Amplitude

Compared to the baseline, a significant decrease was observed in the amplitude of P3 [*F*_(__1, 13__)_ = 12.692, *P* = 0.003], and a significant increase was observed in the amplitude of P2 [*F*_(__1, 13__)_ = 69.357, *P* = 0.000] after TSD. Although the N2 amplitude decreased after 36 h of TSD, the difference did not reach statistical significance (Table 2).

**TABLE 2:** Grand-average peak amplitude of the P2, N2, and P3 components in the correct response condition across multiple electrode sites at baseline and after 36 h-TSD.

		Baseline			36 h-TSD		
		P2	N2	P3	P2	N2	P3
F3	M (SD)	5.80 (3.89)	−3.61 (4.23)	7.94 (3.87)	8.73 (5.55)	−3.50 (5.70)	6.23 (4.40)
Fz	M (SD)	6.43 (3.82)	−4.14 (4.63)	8.30 (4.20)	9.67 (6.12)	−3.99 (6.81)	6.58 (5.25)
F4	M (SD)	5.86 (3.94)	−2.82 (4.29)	8.85 (4.25)	9.38 (5.88)	−2.96 (5.24)	6.59 (5.09)
C3	M (SD)	4.08 (3.08)	−2.37 (4.16)	8.31 (3.09)	6.68 (4.76)	−1.67 (5.44)	6.29 (3.39)
Cz	M (SD)	5.83 (3.63)	−1.89 (5.00)	9.65 (3.78)	8.93 (6.09)	−1.34 (6.23)	7.65 (4.60)
C4	M (SD)	4.43 (3.31)	−0.45 (3.70)	9.87 (3.35)	7.50 (5.00)	−0.35 (4.77)	7.96 (3.80)
P3	M (SD)	**–**	−2.12 (6.17)	8.51 (3.82)	**–**	−1.39 (5.04)	8.21 (3.94)
Pz	M (SD)	**–**	−0.82 (4.13)	9.2 (3.59)	**–**	0.02 (4.43)	8.93 (4.20)
P4	M (SD)	**–**	−0.36 (4.16)	7.93 (3.41)	**–**	0.32 (5.04)	8.47 (3.74)

**FIGURE 4:**
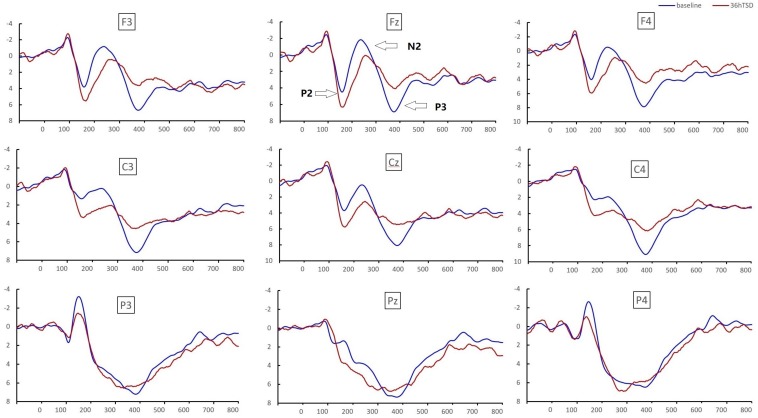
ERP amplitude at baseline and 36 h-TSD for the correct response condition for the working memory task. The channels are ordered from left to right and top to bottom as follows: F3, Fz, F4, C3, Cz, C4, P3, Pz, and P4. Compared to the baseline, the latencies of the N2-P3 components were prolonged, and the amplitudes of N2-P3 were decreased after 36 h-TSD.

**FIGURE 5:**
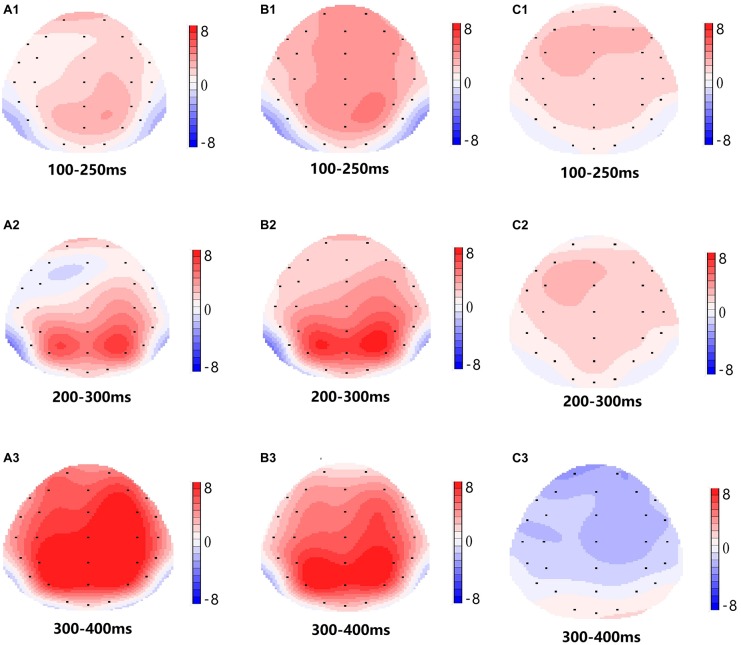
Topographic map of the correct response in the working memory task in different sleep conditions **(A1–C3)**. **(A1)** P2, 100–250 ms, at baseline. **(A2)** N2, 200–300 ms, at baseline. **(A3)** P3, 300–400 ms, at baseline. **(B1)** P2, 100–250 ms, at 36 h-TSD. **(B2)** N2, 200–300 ms, for 36 h-TSD. **(B3)** P3, 300–400 ms, for 36 h-TSD. **(C1)** P2, 100–250 ms, 36 h-TSD with baseline subtracted. **(C2)** N2, 200–300 ms, 36 h-TSD with baseline subtracted. **(C3)** P3, 300–400 ms, 36 h-TSD with baseline subtracted.

Significant main effects of regions and sites on the P2 amplitude were found [*F*_(__1, 13__)_ = 15.889, *P* = 0.002; *F*_(2, 26__)_ = 26.190, *P* = 0.000, respectively] under the TSD condition. During TSD, the maximum amplitude of P2 appeared in the frontal region (Figure 4). In addition, the differences in P2 amplitudes in different regions (frontal vs. central) were more significant [*F*_(__2__, 26__)_ = 8.996, *P* = 0.001] in the bilateral electrodes (left: *P* = 0.001; right: *P* = 0.000) than in the middle electrodes (Figure 4). A significant main effect of the region [*F*_(2, 26)_ = 4.137, *P* = 0.050] and site [*F*_(2, 26)_ = 7.46,*P* = 0.003] on N2 revealed that the N2 amplitude was more negative in the frontal than in the central region (*P* = 0.008, Figure 4) and was smaller on the right than on the left side (*P* = 0.011, Figures 5A2,B2). A main effect of site on the P3 amplitude was observed [*F*_(2, 26)_ = 5.363, *P* = 0.023]. The amplitude of P3 was more positive in the middle than on the left side (*P* = 0.009, Figure 4). A significant interaction effect between time and region was observed for the P3 amplitude [*F*_(2, 26)_ = 7.375, *P* = 0.012]. During TSD, the reduction in P3 amplitude was more significant in the frontal and central regions than in the parietal region (*P* = 0.005; *P* = 0.003) (Figure 5C3). No other main effects or interaction effects reached statistical significance.

### Latency

The latencies of N2 [*F*_(1, 13__)_ = 6.673, *P* = 0.023] and P2 [*F*_(__1, 13__)_ = 8.439, *P* = 0.012] were significantly prolonged after TSD. Although the P3 latency was prolonged after 36 h of TSD, the difference did not reach statistical significance (Table 3).

**TABLE 3:** Grand-average peak latency of the P2, N2, and P3 components in the correct response condition across multiple electrode sites at baseline and after 36 h-TSD.

		Baseline			36 h-TSD		
		P2	N2	P3	P2	N2	P3
F3	M (SD)	164.96 (22.60)	245.05 (27.17)	376.55 (20.25)	173.65 (23.80)	262.01 (40.37)	379.11 (25.86)
Fz	M (SD)	164.71 (22.48)	243.36 (25.49)	374.75 (19.90)	175.11 (22.87)	260.72 (31.55)	382.70 (29.48)
F4	M (SD)	165.87 (23.54)	239.46 (27.33)	373.20 (19.92)	174.66 (24.87)	261.86 (39.24)	380.26 (25.93)
C3	M (SD)	167.69 (26.33)	234.64 (28.98)	370.71 (19.19)	179.20 (27.57)	238.39 (40.16)	363.80 (32.50)
Cz	M (SD)	166.58 (20.32)	236.33 (31.09)	362.17 (24.53)	176.27 (25.83)	238.04 (40.83)	366.19 (42.65)
C4	M (SD)	174.63 (24.57)	230.75 (33.37)	368.18 (18.65)	189.29 (26.59)	242.18 (44.43)	365.59 (35.13)
P3	M (SD)	–	218.71 (54.68)	354.11 (29.25)	–	213.39 (41.47)	357.09 (44.99)
Pz	M (SD)	–	213.57 (37.03)	350.28 (34.14)	–	229.17 (46.15)	341.13 (41.06)
P4	M (SD)	–	221.30 (54.50)	326.72 (38.87)	–	227.36 (50.51)	324.11 (48.03)

The significant main effect of region on N2 [*F*_(2, 26__)_ = 13.789, *P* = 0.001] and P3 [*F*_(2, 26__)_ = 45.226, *P* = 0.000] revealed that the latency of the N2-P3 components was shorter in the parietal region than in the frontal region (*P* = 0.002; *P* = 0.000) and central region (*P* = 0.000; *P* = 0.000) (Figure 4). The latency of the P3 wave was significantly longer on the left side than on the right side [*F*_(2, 26)_ = 8.812, *P* = 0.001] (Figure 4).

No other main effects or interaction effects reached statistical significance.

The N2, P2, and P3 amplitudes and latencies that were elicited at the nine electrode sites are presented in Figure 4. The topographic map of the correct response in the working memory task in different sleep conditions (baseline, 36 h-TSD and the difference between the two conditions) is presented in Figure 5.

## Discussion

In this study, we reported the influences of 36 h sleep deprivation on working memory, combining behavioral data in two sleep states (baseline and 36 h-TSD) with contemporaneous EEG recordings. The analysis of the results indicated that the changes in the behavioral data in accordance with impaired working memory after 36 h TSD: an increase in the mean reaction time of the cognitive tasks and a decrease in accuracy.

Sleep deprivation impaired the individual’s control of attentional resources. Although individuals tried to maintain wakefulness and work performance, including the reaction time and correct rate, during sleep deprivation, the information processing capacity of their working memory was still affected because of the decrease in the speed of processing information (Casement et al., 2006; Wiggins et al., 2018). In this study, the N2 and P3 waves related to working memory were measured to show an increase in latency and a decrease in amplitude after sleep deprivation compared with the baseline readings. Studies have demonstrated that sleep deprivation leads to a continuous decline in attention, and the phenomenon of decreased P3 amplitude indicates that individuals’ top-down control of cognition gradually collapses. Sleep deprivation has a more adverse effect on cognitive functions, especially those that depend on mental or cognitions (Kusztor et al., 2019).

The P3 component reflects the deployment of attention resources, and the latency of P3 is widely seen as the time window for stimulus categorization and evaluation. The decrease in the P3 wave amplitude also confirmed that the decision-making in the matching response after TSD had been damaged to a certain extent (Gosselin et al., 2005). Studies have suggested that sleep deprivation can affect the information processing stage of working memory. In this study, the performance indicators also supported the conclusion that the response time to the target stimulus was increased and that the latency of the P3 wave was prolonged (Cote et al., 2008). It was speculated that the effect of sleep deprivation on P3 components might also take place because of the failure to respond to information alter, which is consistent with previous conclusions that the P3 components are related to the updating of working memory content (Donchin and Fabiani, 1991).

Previous studies have considered the N2 component as an electrophysiological index reflecting the ability of the individual to suppress the response (Kreusch et al., 2014). After sleep deprivation, the prolonged latency of the NoGo-N2 component indicates that the individual’s ability to suppress the response is impaired (Jin et al., 2015). The decreased amplitude and prolonged latency of the N2 component related to pronunciation working memory after sleep deprivation reveals that sleep deprivation impairs the information processing of pronunciation working memory (Zhang et al., 2019). The N2 component is generally thought to reflect the brain’s selective attention and processing of emotional stimuli or signals (Schacht et al., 2008) and is an endogenous component related to an individual’s mental state, attention, and degree of attention. In this study, we found that the latency of the N2 component was significantly prolonged, but the amplitude showed only a downward trend. According to previous studies, prolonged N2 latency reflected an increase in response time after sleep restriction (Zhang et al., 2014). However, the finding that N2 amplitude was not significantly altered may have been due to cerebral compensatory responses (Drummond and Brown, 2001). In the case of limited cognitive resources, there was a compensation mechanism to restore impaired cognitive function (Jin et al., 2015).

According to the scalp topography, the changes in the N2-P3 components related to sleep deprivation are more obvious in the frontal area. Frontoparietal control (FPC) plays an important role in cognitive control. Studies have shown that FPC can bypass top-down cognitive control, enabling individuals to focus on information related to the target while suppressing information that is not related to the target (Smallwood et al., 2011; Wen et al., 2013). FPC is important for information retention and information processing in working memory, and the degree of activation of FPC after sleep deprivation was reduced compared to a normal sleep group (Ma et al., 2014). Although the EEG results did not reflect the changes in specific brain regions in detail, it intuitively reflected the effect of TSD on the retention and processing of working memory information.

Although the exact cognitive process that the P2 component underlies is still widely debated, as a broad definition, the P2 component reflects the process of attention and visual processing and is generally considered to be related to selective attention and working memory, reflecting the early judgment of the perceptual process (Saito et al., 2001). In this study, we found a significant increase in the P2 wave amplitude after sleep deprivation. Studies have reported that P2 waves which might be a part of the early cognitive matching system for message processing and may compare sensory inputs to stored memory (Freunberger et al., 2007) are sensitive to alterations in mission attention and working memory demands (Smith et al., 2002). Functional compensation is one of the unique functions of the human brain and an important factor for maintaining cognitive function. Excessive activation of the dorsolateral prefrontal cortex (DLPFC) after sleep deprivation indicates that, as brain resources decrease, the DLPFC appears to have a compensatory function (Drummond et al., 2004; Choo et al., 2005). Therefore, we speculate that the significant increase in P2 amplitude observed in this study may be due to functional compensation in which individuals appear to maintain normal cognitive function after sleep deprivation. Although a large number of studies have used ERP technology to explore the effect of sleep deprivation on cognitive functions, early components such as N1 and P2 have not been systematically studied, and the results are inconsistent (Evans and Federmeier, 2007; Wiggins et al., 2018; Zhang et al., 2019). There are few researches explore the change of P2 component during sleep deprivation (Mograss et al., 2009). Therefore, the effects of sleep deprivation on early components of ERP, such as P2, still need to be further studied and explored.

In this experiment, we used the 2-back model to design pronunciation, spatial, and object working memory tasks and examined the impairment of working memory after 36 h of TSD. Compared with previous studies that focused only on the effects of sleep deprivation on a specific type of information, such as pronunciation working memory, or specific cognitive function, such as response inhibition, we have considered the contents of the working memory model and comprehensively analyzed the effects of sleep deprivation on working memory.

However, the study has some limitations. First, we only used the 2-back task and failed to compare the performance of the participants in working memory tasks of different difficulties. Therefore, there are limitations in explaining and inferring changes in workload. Second, only male volunteers were used in the study, and the conclusions need to be assessed when extending them to female volunteers. Due to the limited number of participants, we found only that the amplitude of the N2 wave had a downward trend and that the P3 wave latency had a prolonged trend. Stable results might be obtained after increasing the number of participants. Third, combining our procedure with fMRI for working memory may facilitate further interpretation of the results. Previous studies have shown that circadian biorhythms affect behavioral performance, and there are individual differences (Montplaisir, 1981; Lavie, 2001). We did not record the EEG data at the same time point in this experimental, so the influence of circadian biorhythms on the test results cannot be completely ruled out.

This research showed that working memory ability was impaired after TSD and that this damage was not associated with the stimulus content of working memory. The lack of sleep reduced the quality of the information stored in memory, which might occur with the degenerative process of attention (Ratcliff and Van Dongen, 2018). This study provides electrophysiology evidence for understanding the mechanism under the impaired working memory after sleep deprivation. It is necessary to pay attention to the adverse effects of working memory impairment caused by sleep deprivation and to explore effective interventions for such damage.

## Data Availability Statement

The datasets generated for this study are available on request to the corresponding author.

## Ethics Statement

The studies involving human participants were reviewed and approved by The Fourth Military Medical University Beihang University. The patients/participants provided their written informed consent to participate in this study.

## Author Contributions

YS designed the experiments. ZP produced the results and wrote the manuscript. CD and LZ analyzed and interpreted the data. JT and YS performed the experiments, acquainted the data, and the guarantors of this study. YB, LZ, and JT contributed to participating in data collection and reviewing the literature. All authors listed have read and approved the final manuscript.

## Conflict of Interest

The authors declare that the research was conducted in the absence of any commercial or financial relationships that could be construed as a potential conflict of interest.
